# Generation, evolution, interfering factors, applications, and challenges of patient-derived xenograft models in immunodeficient mice

**DOI:** 10.1186/s12935-023-02953-3

**Published:** 2023-06-21

**Authors:** Mingtang Zeng, Zijing Ruan, Jiaxi Tang, Maozhu Liu, Chengji Hu, Ping Fan, Xinhua Dai

**Affiliations:** 1 Department of Pharmacy, West China Hospital, Sichuan University, Chengdu, 610041 China; 2 Department of Laboratory Medicine, West China Hospital, Sichuan University, Chengdu, 610041 China

**Keywords:** Patient-derived xenograft, Preclinical model, Cancer research, Immunotherapy, Immunodeficiency mice, Humanized mice

## Abstract

Establishing appropriate preclinical models is essential for cancer research. Evidence suggests that cancer is a highly heterogeneous disease. This follows the growing use of cancer models in cancer research to avoid these differences between xenograft tumor models and patient tumors. In recent years, a patient-derived xenograft (PDX) tumor model has been actively generated and applied, which preserves both cell–cell interactions and the microenvironment of tumors by directly transplanting cancer tissue from tumors into immunodeficient mice. In addition to this, the advent of alternative hosts, such as zebrafish hosts, or in vitro models (organoids and microfluidics), has also facilitated the advancement of cancer research. However, they still have a long way to go before they become reliable models. The development of immunodeficient mice has enabled PDX to become more mature and radiate new vitality. As one of the most reliable and standard preclinical models, the PDX model in immunodeficient mice (PDX-IM) exerts important effects in drug screening, biomarker development, personalized medicine, co-clinical trials, and immunotherapy. Here, we focus on the development procedures and application of PDX-IM in detail, summarize the implications that the evolution of immunodeficient mice has brought to PDX-IM, and cover the key issues in developing PDX-IM in preclinical studies.

## Introduction

Cancer, as a disease affecting human life and health worldwide, has been of high concern [1]. Among various treatments for cancer, chemotherapy has become an increasingly mature therapy, unfortunately the efficacy has been discounted. In the early preclinical stage for developing new therapies, it is necessary to adopt appropriate in vitro or in vivo preclinical models to estimate the efficacy and possible toxicity of anticancer drugs to cancer patients [2]. Current tumor models for drug evaluation generally are to implant xenografts derived from well-established human cancer cell lines into immunodeficient mice. However, cell line models not only lack heterogeneity and tissue structure, but also fail to accurately mimic the complex tumor environment involving tumor deterioration under hypoxic conditions, excessive hypoxia-induced transcription factor activation, defective immune evasion mechanisms and angiogenesis [3]. Therefore, cell line models might not be the most appropriate models for evaluating the efficacy of novel drugs.

As a possible solution, xenografts derived from engrafting fresh surgical specimens directly into immunodeficient mice have facilitated the development of in vivo models of human tumors [4]. Such patient-derived xenograft models (PDXs) established by direct metastasis of tumor tissue retain morphological, structural, and molecular characteristics similar to those of primary cancer [5]. The interaction from host microenvironment, genetic characteristics, gene expression patterns, and histological characteristics of the original patient were reproduced in immunodeficient mice that have received transplants [6, 7]. The above characteristics will directly support PDXs as a reliable strategy to anticipate clinical findings and rapidly screen potential therapies, which provides guidance for optimizing personalized treatment in advanced cancer, and suggest new treatment opportunities for patients without other treatment options [8].

In recent years, many attempts have been made to provide additional models, such as genetically engineered mouse models (GEMMs), alteration of the host in xenograft models (zebrafish), innovations in culture patterns (conditionally reprogrammed cell cultures and induced pluripotent stem cells, etc.), and several in vitro models (organoids, spheroids, and microfluidics) [9–12]. These platforms have their own characteristics in terms of tumor architecture, microenvironment, cellular composition and heterogeneity, stem differentiation status, growth patterns, and response to treatment. However, partially significant limitations have led to low scoring of these traditional models in the evaluation criteria for animal models (Table 1). They still have a long way to go to become reliable models leading to a better understanding of fundamental cancer biology and future applications to translational cancer research. PDX models established in immunodeficient mice (PDXs-IM), as one of the most reliable and standard models in preclinical studies approximately a century after the first tumor model, are more expected to effectively connect nonclinical and clinical data in translational research, ultimately becoming the standard "Avatar" model for human cancer research.

**Table 1:** Characteristics of different cancer models

Models	Advantages	Limitations	Recommendations
PDX model in immunodeficient mice	(1) Highly recapitulate tumor microenvironment (2) High fidelity (3) High predictive value (4) Can be used in metastasis models	(1) Unsatisfactory take rate (2) High cost (3) Technically challenging	(1) Developing new immunodeficient mice (2) Determining the most appropriate conditions and methods to improve the take rate
PDX model in humanized immunodeficient mice	(1) Can be evaluated for immunotherapy (2) Partly recapitulate tumor microenvironment	Therapeutic effect is affected by immunity	Developing more comprehensive and functional immune system humanized mice
PDX model in zebrafish	(1) Transparent embryos facilitate the visualization of tumor processes and the tracking of fluorescently labeled cells (2) Easy to culture, low cost (3) Allow large-scale and high-throughput screening	(1) Not a mammal (2) The rate of proliferation or the manner in which the transplanted human cells formed tumor masses differed from those of immunodeficient mice or human patients	Standardization of zebrafish xenograft techniques and application modalities
Cell line-derived xenograft model	(1) Low cost, easy establishment and high take rate (2) Slightly recapitulate tumor microenvironment	(1) Can’t reproduce heterogeneity (2) Can’t maintain the original cell properties (3) Low predictive value	Large numbers of mice were used at relatively early stages of drug development to reflect heterogeneity among tumors
Circulating tumor cell-derived xenograft model	(1) High heterogeneity in the metastatic environment (2) High tumorigenicity (3) Partly recapitulate tumor microenvironment	(1) Difficulty in the isolation and counting of CTCs (2) Organ metastasis is affected by the injection site of CTCs	(1) Capturing the heterogeneity of CTCs by drug sensitivity assay (2) In vitro culture of CTCs
Patient-derived organoid model	(1) No ethical issues (2) Time saving, suitable for high-throughput drug screening (3) Maintenance of the gene expression profile of the initial tumor over an extended period of time (4) Available for low malignant tumors	(1) Lack of uniform standards (2) Tumor free microenvironment (3) Limited tissue availability (4) Labour intensive	(1) Develop high-throughput systems (2) Development of uniform modeling standards (3) Evolution of co-culture models (4) Increased access to biobanks (5) Use of circulating tumor cells
Genetically engineered mouse model	(1) Recapitulate tumor initiation and early development process (2) Specific genes can be studied in detail (3) Can be increased easily after establishment	(1) Can’t reproduce heterogeneity of human tumor (2) Take a long time to be established (3) Low predictive value	Investigating how a specific gene of interest could contribute to tumor initiation and relapse

With the development of highly immunocompromised mouse receptors, PDXs-IM have been successfully constructed by domestic and foreign research institutions including breast cancer, liver cancer, pancreatic cancer, esophageal cancer, gastric cancer, colorectal cancer, cervical carcinoma, bladder cancer, non-small cell lung cancer, pleural mesothelioma, squamous cell carcinoma of head and neck, glioblastoma and small cell lung cancer, etc. [13]. In this review, we will describe in detail the impact of the evolution of immunodeficient mice on PDX-IM, elucidate the points to consider when establishing such models, and investigate the application of PDX-IM models to cancer research.

## Patient-derived xenograft models in immunodeficiency mice

**Fig. 1:**
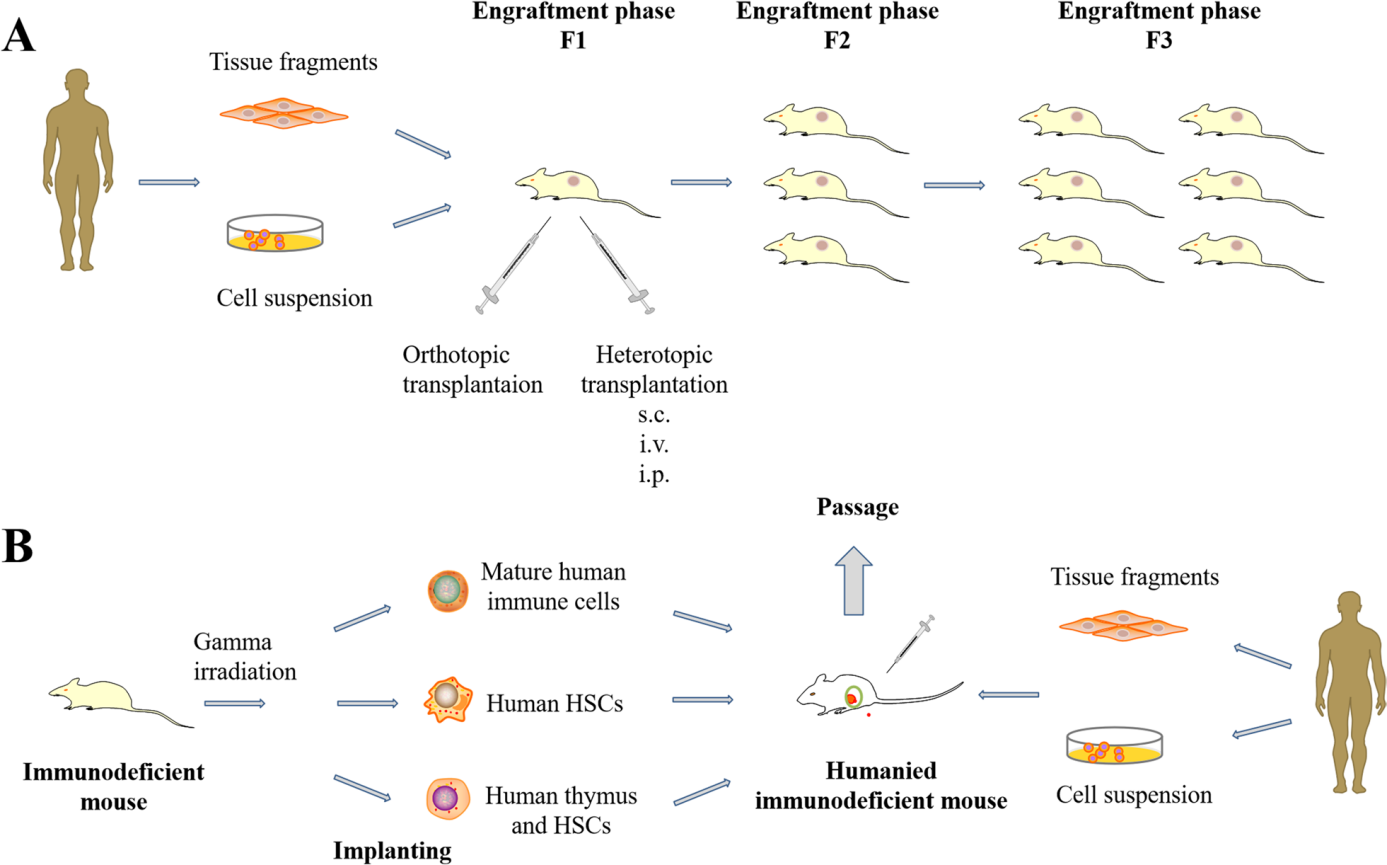
The process of creating PDX models in immunodeficient mice (**A**) and human immunodeficient mice (**B**)

Tumor biopsies or tissues from patients were implanted into immunodeficient mice to generate PDX-IM models that better reflect the tumor-stromal interactions present in the primary tumors (Fig. 1A), although the matrix is derived from the host [14]. In contrast to cell line-derived xenograft models, the PDX-IM model has great potential for efficacy assessment and co-clinical studies. Previous studies have identified that tumors formed in PDX-IM models are histologically and genetically similar to patients' original tumors, in addition to their high genomic fidelity [15]. For example, Dong et al. established 33 RCC mouse xenograft models by biopsy transplantation and tumor resection transplantation, accompanied by differences in transplantation rates between the two approaches. The PDX-IM tumors were highly consistent with the primary tumors in regard to histology, mutation status, copy number changes and targeted therapy response [16]. Furthermore, fifteen PDX-IM models were successfully established from 62 gastric cancer patients and passaged to maintain tumors in immune-compromised mice. The histological and genetic characteristics of PDX-IM models were relatively stable in passage through the comparison of genomes and other characteristics of later generations [17]. Despite some drawbacks, including low transplant rates and high costs, the PDX-IM model has been widely used in personalized medicine, drug screening, combination clinical therapy, and especially in drug efficacy prediction. In terms of metastatic models, cell line-derived xenograft models, as well as transgenic mouse models, often fail to reproduce key mechanisms. Patient-derived xenograft models have become an attractive alternative as they more effectively reflect the diversity and heterogeneity of tumors, while considerable progress has been made in metastasis research [18]. Therefore, the PDX-IM model is currently the most powerful tool for assessing tumor-related mechanisms. The success rate of grafting primary tumor specimens into PDX-IM models is affected by the type of immunodeficient mice, as detailed later in the review.

## Patient-derived xenograft models in Humanized immunodeficiency mice

With the rapid development of cancer immunotherapy in recent years, in addition to the models for screening chemotherapeutic drugs, researchers also need a PDX-IM model that can be used for immunotherapy evaluation, known as humanized PDX-IM model [19]. This model is currently the best pre-clinical model for evaluating immunotherapy, providing numerous insights into the behavior of different cancers in their own tumor microenvironment under the action of human immune cells. This necessitates the creation of mice with a human immune system on the basis of immunodeficient mice as a vehicle to better test the efficacy of immunotherapy (Table 2) [20]. In general, human derived mice that contributed to the establishment of the humanized PDX-IM model by combining with the PDX-IM model (Fig. 1B), can be obtained by injecting peripheral blood mononuclear cells (PBMC) or CD34^+^ human hematopoietic stem cells (HSCs) (obtained from bone marrow, cord blood, fetal liver or thymus tissue) [21]. However, PBMCs may cause severe graft-versus-host responses, meanwhile obtaining CD34^+^ cells from clinical cancer patients is challenging [22]. At present, several strains of immunodeficient mice have been utilized to produce humanized mice: the NOD.Cg-PrkdcscidIl2rgtm1WjlTg (CMV IL-3, CSF2, KITLG)1Eav/MloySzJ (also known as NSG-SGM3) mice, the NOD, B6. SCID Il2rγ − / − KitW41/W41(NBSGW) mice and the human SIRPA and IL15 knockin(SRG-15) mice [23–25]. Unfortunately, various deficiencies remain in the humanized mice, in particular different degrees of immune system reconstruction, which may lead to different efficacy of immunotherapy. Several types of human hematopoietic cells could not sufficiently differentiate with hematopoietic stem cells in any humanized mouse strain, such as erythrocytes, platelets, neutrophils, NKT cells, and ILC2 [20]. In addition, conventional humanized mouse models suffer from incomplete replacement of the hemato-lymphoid system and inefficient myelopoiesis in humans [26].

**Table 2:** Different types, construction methods, characteristics, and applications of humanized mice

Mouse strain	Types of methods	Specific operation process	Advantage	Shortcoming	Immunotherapeutic applications
NOD/SCID IL-2Rγ C (NSG) BALB/C Rag2 IL-2R γ C (BRG)	Humanized-PBMCs/Humanized-PBLs	Intravenous injection of PBMCs (5–10 × 10^6^)	1. Cost effective; 2. Simple establishment; 3. Suitable for T-cell-related immune research	1. B, NK, and other immune cells fail to proliferate in vivo; 2. GVHD development; 3. EBV-associated lymphoproliferative; 4. Xenograft rejection	1. Adoptive NK and T cell therapy; 2. Tumor microenvironment evaluation; 3. CAR-T and NK cell therapy; 4. Immune check point inhibitor investigation; 5. Tumor-Infiltrating Lymphocyte therapy; 6. Gene therapy; 7. Dendritic cell therapy; 8. Targeted therapy; 9. Evaluation of microbiota-associated cancer treatment
NOG, NSG, NOD/SCID, BRG	Humanized-HSCs (CD34^+^)	Intravenous injection of 1 × 10^5^ HSCs	1. More complete immune reconstitution; 2. GVHD rarely occurs	1. Lack of T cells; 2. Limited sample sources	
NOG, NSG, NOD/SCID, BRG	Humanized-BLT	Intravenous injection of CD34^+^ HSC (0.5–1 × 10^6^) from human bone marrow, implantation of human fetal liver and thymus in to mouse sub renal capsule	1. Human T cells are restricted to human HLA; 2. Higher immune reconstitution; 3. Long term existence of model	1. GVHD development; 2. Engraftments should be carried from the same donor; 3. Complex technique and ethical problems; 4. Limited sample sources	
MI(S)TRG, NSG	Genetic engineering	Human immune genes are knocked into respective mouse loci	Approximating the levels in the human system	Complex technique and expensive	

*PBMCs* peripheral blood mononuclear cells, *PBLs* peripheral blood lymphocytes, *HSCs* hematopoietic stem cells, *BLT* bone marrow, *liver* thymus

This status has been partially improved by the advent of cytokine humanized mice (MISTRG mice), which combine genetic preconditioning and cytokine-mediated support by knocking in gene replacement, removing mouse cytokine-encoding genes and replacing them with their human counterparts [24, 27]. In these mice, there was a clear increase in the level of human hematopoietic engraftment in organs. For example, human phenotypically defined heat shock protein cells in the bone marrow, T cells in the thymus, and myeloid cells in non-hematopoietic organs have elevated levels of engraftment that approach those in the human system [28]. In this way, the innate and adaptive immune responses to diseases such as COVID-19 and myelodysplastic syndromes in humans have been faithfully recapitulated [29, 30]. Radtke et al. developed the first "monkeyized" mouse xenografts through the MISTRG mouse model, which allowed for pre-evaluation of novel HSC-mediated gene therapies, thereby enabling more facile and fewer costly evaluation of promising strategies [31]. In addition, the establishment of a mouse model of humanized immune system has been further improved: such as irradiation or chemical pretreatment, depletion of auto-immune cells in mice, injection of human cytokines, construction of viral vectors, and injection of gene expression plasmids.

## Development of immunodeficient mice

Immune-deficient mice implanted with the human immune system provide powerful models for the study of human immunology in vivo, and the PDX-IM model using these mice is a critical tool for discussing the interaction of human immunity with various cancers. Therefore, the use of the most appropriate host mouse strain to generate PDX-IM models is an essential consideration, with different applications and research benefits in different tumors. At present, several types of immunodeficient mice can be used to establish xenograft models: nude mice, severe combined immunodeficient (SCID) mice, SCID/Beige mice, non-obese diabetic (NOD) mice, NOD/SCID mice, NSG mice, BALB/c mice, etc. (Table 3). Studies have reported that NOD/SCID mice are mainly used for lung cancer and melanoma, NSG mice for breast, SCCHN and ovarian cancer, Balb/c nude mice for colon, pancreatic and gastric cancer and renal cell cancer, and SCID mice for prostate cancer [32].

**Table 3:** Strains of immunocompromised mice used to develop the PDX-IM model

Mouse strain	Origin	Phenotype	Advantage	Shortcoming	Engraftment
Nude	Homozygous mutations in the Foxn1 gene	1. Thymus and hair are absent; 2. Antigen-presenting cells, macrophages, and NK cells are active	1. Well characterized; 2. Easy observation of subcutaneous tumors; 3. Low price	1. Functional B and NK cells; 2. T cell leakage increases with age	1. Low efficiency; 2. Not an ideal host
Rag1/Rag2	Rag1/2 recombinase defects	No mature T and B cells	Easy to evaluate DNA damaging therapies	1. NK cell activity is high; 2. Restrict HSC reconstitution in humans	Low efficiency
SCID	Mutations in the Prkdc gene	No mature T and B cells	Higher engraftment rate compared to nude	1. Functional NK cell; 2. Leakage of T and B cells; 3. The probability of death is very high; 4. Radiosensitive	1. Low efficiency; 2. Manily for prostate cancer
SCID/Beige	Combination of Beige mutation with the SCID mutation	1. No mature T and B cells; 2. Impaired Mφ and NK function; 3. Macrophage increase	Higher engraftment rate compared to SCID	1. Leakage of T cells; 2. Radiosensitive	1. Moderate efficiency; 2. Commonly used in F1
NOD/SCID	NOD (non-obese diabetic) mutation along with SCID	1. No mature T and B cells; 2. Impaired NK function and Mφ & DC	Better engraftment	1. Spontaneous lymphoma; 2. Short life span (av. 36 wks); 3. Radiosensitive	1. Moderate efficiency; 2. Manily for lung cancer and melanoma
NOD/SCID/IL2Rγnull (NOG/NSG)	NOD/SCID mice with IL2/ receptor gamma truncation/disruption mutations	1. No mature T, B cells, and NK cells; 2. Impaired Mφ and DC	Excellent engraftment of PDX-IM including hematopoietic malignancies	1. Need strict SPF conditions; 2. Breeding is not easy; 3. Expensive	1. High efficiency; 2. Manily for breast, SCCHN and ovarian cancer
NOD/SCID/Jak3null (NOJ)	Backcrossing JAK3null mice with the NOD.Cg-Prkdcscid strain	1. No mature T, B cells, and NK cells; 2. Impaired Mφ and DC	1. Low incidence rate of lymphoma; 2. Longer life spans; 3. Suitable for low-growth tumors	1. Need strict SPF conditions; 2. Breeding is not easy; 3. Expensive	1. High efficiency; 2. Manily for breast, SCCHN and ovarian cancer
NOD-SCID-IL2RG − / − (NRG)	Rag1 mutation replaced the SCID mutation in NOG mice	1. No mature T, B cells, and NK cells; 2. Impaired Mφ and DC	1. Intra-oral injection for PDX-IM development; 2. Longer life spans (> 90 wks); 3. Low incidence rate of lymphoma	1. Macrophages, DCs and neutrophils influence engraftment efficacy; 2. GVHD development	1. High efficiency; 2. Manily for hormone receptor-positive (HR +) breast cancer; 3. Manily for human HSC engraftment
BALB/c Rag2null/IL2Rγnull (BRG) Rag2 null/Jak3 null (BRJ) BRGSF	BALB/c mice with Rag2/IL2rg/Jak3 receptor gamma truncation/disruption mutations	No mature T, B cells, and NK cells	1. Excellent engraftment of PDX-IM; 2. Resistant to stress; 3. Easy breeding; 4. Resistance to radiation	Expensive	1. High efficiency; 2. Manily for cholangiocarcinoma, head and neck tumor, gastric cancer, bladder cancer, and hematological tumor

*NK* natural killer cells, *Mφ* macrophages, *DCs* dendritic cells, *SCCHN* squamous cell carcinoma of the head and neck, *GVHD* chronic graft versus host disease, *HSC* hematopoietic stem cell

Nude mice have been used as recipients of human tumor xenograft, accompanied by high implantation rate of gastrointestinal tumors, easy observation of subcutaneous tumors and low price, thus they are still an important resource for PDX-IMs establishment with an efficiency of 75% [3, 33, 34]. However, because the complete (or activated) innate immunity and leakage of T cells in nude mice restrict the options for human cancer transplantation, SCID mice have been developed to improve the efficiency of tumor transplantation [35, 36]. Affected by the function of remnant natural killer (NK) cells that prevent homing and maintenance of human cells, transplantation efficiencies of human blood cells and tumor cells in this mouse models are not as high as expected [37]. Fortunately, SCID/Beige mice were established by crossing SCID mice with Beige mice to overcome the effects of NK cells, increase uptake of human tumor cells, and are more commonly used in F1 [38].

NOD/SCID mice with IL2rg mutations, such as NOD.Cg-PrkdcscidIl2rgtm1Wjl (NSG) or NODShi.Cg-PrkdcscidIl2rgtm1Sug (NOG) mice, have highly enhanced immunodeficiency and are able to engraft almost all types of human cancers [39]. Moreover, NOD.Cg‐PrkdcscidIl2rgtm1Sug/Jic, NOD.Cg‐PrkdcscidIl2rgtm1Wjl/SzJ and NOD.Cg‐PrkdcscidJak3tm1card are also the major tools for establishing PDX-IMs [14, 33], which is characterized by a higher degree of immunodeficiency due to the decline or complete absence of natural killer (NK) cell function [33]. For hematological malignancies, such as leukemia and multiple myeloma, it is necessary to implant them directly into the blood or bone marrow of NOG/NSG mice. The BRJ mice have been used as alternative recipients of cholangiocarcinoma PDX-IMs with a high engraftment ratio (75%) [39, 40]. Additional solid tumors, such as head and neck tumors, gastric cancers, and bladder cancers, were also transplanted into the BRJ mice with relatively high engraftment rates compared to currently available models. Since BRJ mice are easy to breed and maintain, and have the benefits of both BRJ and nude mice, they may be ideal models for passaging and drug evaluation [41]. This, in part, explains the importance of selecting mouse strains for cancer research as the number of immunodeficient strains increases.

## Interfering factors to consider of establishing PDX-IM model

**Fig. 2:**
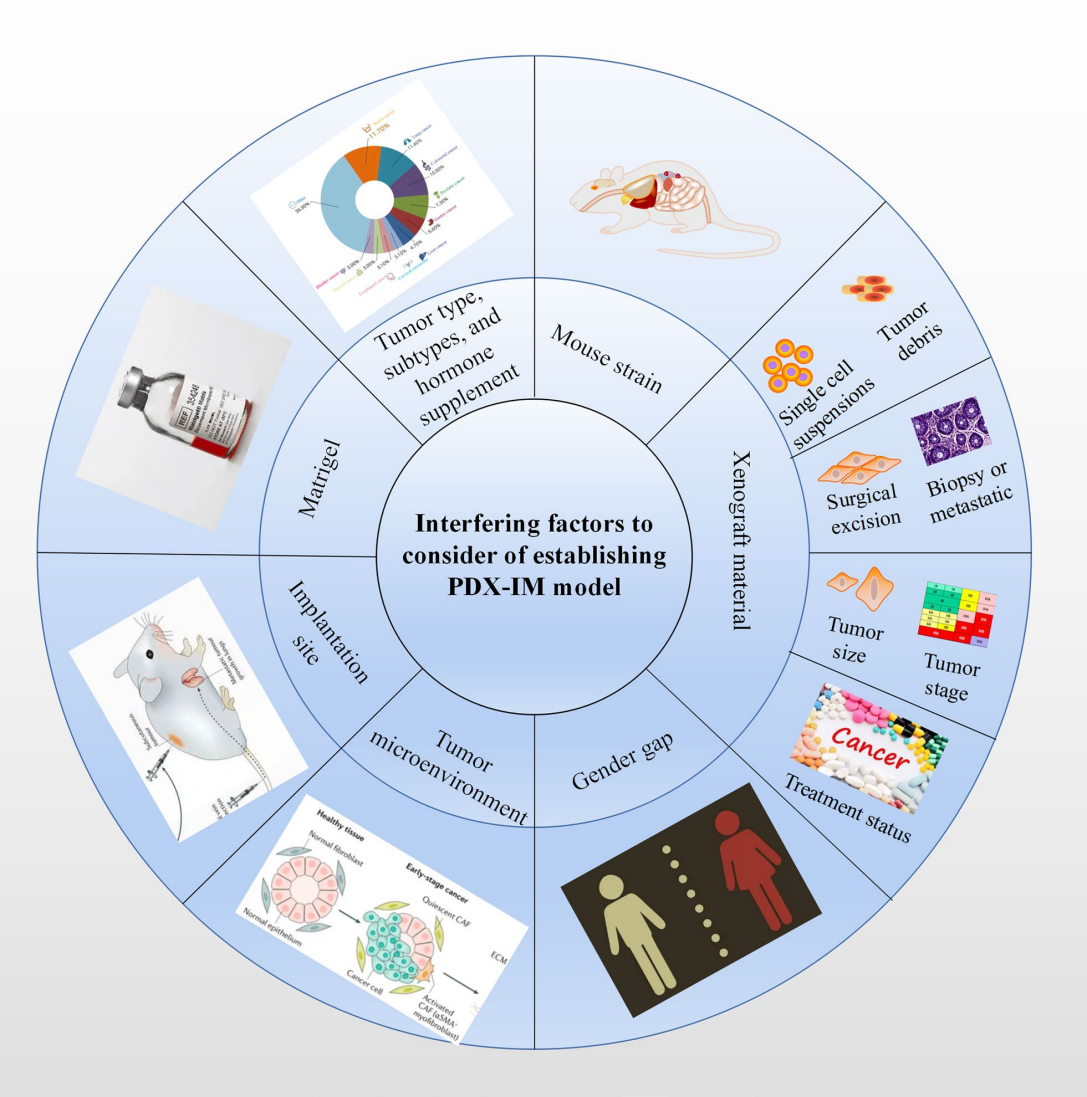
The interfering factors to consider of establishing PDX-IM model, including mouse strain, tumor type, subtypes, hormone supplement, tumor microenvironment, Matrigle, xenograft material (tumor debris, single-cell suspensions, surgical excision, biopsy or metastatic samples, tumor stage and size, treatment status), implantation site, and gender gap

The PDX-IM model is promising and has led to some exciting breakthroughs in oncology research. However, not all patient tissues can be successfully established as PDX-IM models, and the obstacles that hinder the establishment of PDX-IM model normalization still deserve to be considered. In addition to mouse strain, several factors (Fig. 2), such as (a) tumor type, subtypes, and hormone supplement, (b) tumor microenvironment, (c) Matrigle, (d) xenograft material, (e) implantation site, and (f) gender gap need to be investigated. In order to better utilize PDX-IM models, effective establishment of them is particularly important.

### Tumor type, subtypes, and hormone supplement

The success rate of establishing PDX-IMs varies depending on the source of the tumor and the characteristics of the disease, such as tumor invasiveness, relapse/recurrence status, primary or metastatic tumors, and higher transplantation rates tend to manifest in more aggressive, recurrent, and highly metastatic tumors (Table 4). The reported success rates of PDX-IM models generally range from 23 to 75% [42]. Gastrointestinal cancer, such as colon cancer (64–89%) and pancreatic cancer (62%), seems to have higher transplant rates than other cancers. However, the response rate varies greatly among different subtypes of gastric cancer [43]. The success rate of breast cancer implantation is very low (13–27%), especially for estrogen receptor-positive breast cancer, even in the most successful laboratory of PDX-IMs engraftment [44]. Among human breast cancers, triple-negative breast cancer has the highest acceptance rate (51.3%), followed by HER2-positive (26.5%) and luminal B (5.0%). Furthermore, the stability rate of ER-negative and PR-negative (52% and 37%) was significantly higher than that of ER-positive and PR-positive tumors (2% and 3%) [45]. In general, orthotropic transplantation is required for this cancer model [14]. For hormone-dependent tumors, the success of PDX-IM model transplantation is also affected by the experimental protocol, and human hormone replacement therapy can be used to improve the transplantation rate [46]. Most ER-positive PDX-IM models of breast cancer remain estrogen-dependent in tumor growth, and their response to endocrine therapy is consistent with the clinical response of tumor origin. Supplementation of estradiol pellets increased the stability rate of xenografts from 2.6% to 21.4% [42]. In prostate cancer, the development of the prostate cancer xenograft model has been hampered by low success rates, in part by the poor vascularization of the transplantation site, which is successful only when applied to advanced cancers with high growth rates [47]. Supplementation of exogenous androgen shortened the latency of tumorigenesis and improved the rate of tumorigenesis [48]. Lin et al. supplemented testosterone in non-obese diabetic (NOD)/SCID male mice, resulting in a 44% transplantation success rate [47].

**Table 4:** Current status of transplantation based on multiple interfering factors in different tumors

Tumor type	Mice strain	Sample source	Subtype	Xenograft material	Implantation site	Engraftment ratio (%)	RF
Cholangiocarcinoma	NOD/SCID	Surgical resection	–	4 × 4 mm	s.c.^*^	5.8	[93]
	BRJ	Surgical resection	ICC, ECC	8–27 mm^3^	s.c	75	[40]
Breast cancer	Nude	Surgical resection	ER +	2–3 × 2–3 mm	fat pad^**^	2.5	[94]
			ER −			24.3	
	SCID/Beige	Biopsies	ER +	1 mm^3^	s.c	19	[68]
	NSG		ER −			31.3	
	NOD	–	Primary tumors	5 mm^3^	s.c	100	[95]
			Metastasis model	1 × 10^6^ Cell suspensions	fat pad (orthotopically injected (tail vein)	57	
	NOD-SCID /NSG	Surgical resection	–	8 mm^3^	s.c	27.4	[45]
Pancreatic cancer	SCID	Surgical resection	Pancreatic ductal adenocarcinomas	2–3 mm	s.c	67	[96]
	NSG			1–3 mm^3^		71.1	[97]
	Nude			0.5 mm^3^		44.8	[98]
Gastric cancer	BALB/C	Surgical resection	–	–	s.c	28.1	[99]
	NOD/SCID	Biopsies		2 × 2 × 2 mm^3^		34.1	[88]
	Nude/SCID	Surgical resection		2 mm^3^		16.9/26.9	[100]
	Nude/NOG	Surgical resection		3 × 3 × 3 mm^3^		24.2	[17]
	Nude	Surgical resection		3 × 3 × 3 mm^3^		94	[101]
Colorectal cancer	NMRI/NOG	Surgical resection	–	Smaller fragments	s.c	60	[102]
	Nude/NSG	Surgical resection		–		52	[103]
	NSG	–		2–3 mm^3^		100	[104]
	NSG	Surgical resection		< 150 μm (50% Matrigel)		89.9	[105]
Lung cancer	NOD/SCID	Surgical resection/Biopsies	NSCLC	2–3 mm^3^	s.c	35	[79]
	NSG	Surgical resection	NSCLC	2 mm (10% Matrigel)	s.c	29	[106]
	Nude	Surgical resection	NSCLC	25–30 mm^3^	s.c	30–40	[107]
	NOD/SCID	Surgical resection	NSCLC		r.c	90	
	NSG	EBUS-TBNA	NSCLC	1.0 × 10^5^ Cell suspensions (10% Matrigel)	s.c	42.1	[108]
	NSG		SCLC		s.c	67	
Ovarian cancer	Nude	–	–	–	s.c	19.8	[109]
	BALB/C	Surgical resection	EOC	< 2–3 mm	r.c.^***^	48.8	[110]
	SCID	Surgical resection	–	0.3–0.5 cm^3^ tumor slurry (50% McCoy's media)	s.c	74	[111]
	NSG	Surgical resection	HG-SOC	1–3 mm^3^/ < 1 mm^3^	s.c./intra-ovarian bursal	83	[112]
	NSG	–	–	–	i.p.^****^	31	[113]
	BALB/SCID	Surgical resection	EOC/NOC	–/3 mm^3^	s.c./ovarian in situ (OIS)	18.52	[114]
Head and neck cancer	Nude/NOG	Surgical resection/Biopsies	HNSCC	3 × 3 × 3 mm^3^	s.c	24.2	[115]
	NSG	Surgical resection	HPV + HNSCC	–	s.c	64	[116]
Glioblastoma	NSG	Surgical resection	MB, ATRT, HGG, EPN	10^5^ Cell suspensions	orthotopic	30	[117]
Prostate cancer	Nude	rPE	–	2 × 2 × 1 mm	r.c	39	[118]
	NOD/SCID			1 × 2 × 1 mm	orthotopic	48	
	NSG/NOG	rPE/TUR-P	–	4–5 mm/2–3 mm (testosterone)	s.c./r.c	37	[70]
	SCID	TAN	LuCaP	3–4 mm	s.c	10	[119]
Melanoma	NOG	Biopsies	Stage III and IV metastatic	Cell suspensions (50% Matrigel)	s.c	88.4	[120]
	NSG	Biopsies	–	Fragments (100 μL Matrigel)	s.c	65.8	[5]
Renal cell carcinoma	Nude	Surgical resection	–	5 mm^3^	s.c./orthotopic	8.9	[121]
	NOD/SCID	Surgical resection	–	2–3 mm	r.c	37.2	[77]
	NSG	Biopsies	–	8- 27 mm^3^/1–6 × 10^6^ cells (50% Matrigel)	s.c	45	[122]
	Nude	Surgical resection	Nephroblastoma	1 × 3 × 3 mm^3^	r.c	67	[123]
Medulloblastoma	SCID	Surgical resection	SFRP, WIF1, NPR3, KCNA	Tumor cells (1 × 10^5^)	orthotopic	52	[124]
Cervical Cancer	NOD/SCID	Biopsies	Cancer, dysplasia, and normal cervical tissues	1 mm^3^	r.c	71.4	[125]
Malignant Pleural Mesothelioma	NOD/SCID	Extrapleural pneumonectomy, decortication, or biopsy	–	1 mm^3^	s.c	40	[126]

s.c.* subcutaneous, fat pad** mammarian fat pad, r.c.*** renal capsule, i.p.**** intraperitoneal, *NOC* epithelial ovarian cancer, *tAN* tissue acquisition necropsy

### Tumor microenvironment

The tumor microenvironment is the internal environment in which tumor cells are produced and live. In addition to cancer cells, the microenvironment includes surrounding lymphatics and capillaries, stromal cells (immune cells and cancer-associated fibroblasts), additional normal cells, extracellular matrix (ECM) and various signaling molecules. For the growth and maintenance of cancer cells, changes in microenvironmental conditions play an irreplaceable role [49], involving the promotion of unrestricted cell proliferation, tumorigenesis and direct metastasis of tumors [50]. Among these components of the tumor microenvironment, stromal cells are able to directly regulate the behavior of tumor cells. Tumor growth is accompanied by tumor-specific T-cell maturation and tumor-specific T-cell activation under normal conditions, and accumulation of NK cells is also observed at the tumor site. Tauriello et al. reported that several driver mutations in a mouse model of colorectal cancer were specifically modified in intestinal stem cells to develop metastatic tumors. The quadruple-mutant mice exhibited hallmarks of human colorectal cancer, including T-cell exclusion and TGFb-activated stromal cells. Inhibition of TGF β induced a cytotoxic T-cell response to tumor cells, thus preventing metastasis [51].

Similarly, cancer-associated fibroblasts (CAFs), as one of the most important components of the tumor microenvironment, secrete a variety of cytokines to facilitate tumor growth. Ohlund and his colleagues found that pancreatic stellate cells (PSCs) first differentiate into CAFs and then form stroma. Interestingly, these pancreatic stellate cells have two subtypes: the former could increase the expression of α-smooth muscle actin (α SMA) in tumor cells adjacent to mouse and human PDA tissues, while the latter is located far away from tumor cells and secreting IL-6 and other inflammatory mediators, but lacking α SMA expression. In accordance with this, Seino and colleagues established a co-culture of PDAC organoids and CAFs, indicated that the CAFs provide a WNT niche for PDAC [52], which provides direct evidence for the heterogeneity of CAFs in PDA tumor biology and highlights the importance of CAFs in the tumor microenvironment [53].

Abnormal tumor vascular function, including irregular and premature vascular networks, inadequate microcirculation, and high vascular permeability, may also contribute to the formation of an adverse pathophysiological tumor microenvironment. Hypoxia, in particular, is a common condition in most tumor masses, generally resulting in mutations, inhibition of apoptosis, and epithelial-mesenchymal transition [54]. Furthermore, exosomes produced by cancer cells have been proved to be an active communication mechanism between tumors and their microenvironment, making some breakthroughs in the treatment of drug resistance, metastasis and immunosuppression [55]. Given the high complexity of the composition of the tumor microenvironment, numerous microenvironmental factors should also be considered in the modeling.

### Matrigel

During the establishment of PDX-IM model, a mouse basement membrane extract (matrix gel) was used to improve the xenograft rate by combining with patient-derived biopsy materials. The procedure is simple and requires only attention to hold the needle in place for a few seconds after injection to allow the mixture to condense and prevent leakage. Fridman et al. directly mixed tumor cells with BME/ Matrigel at low temperature and injected to increase uptake and growth of cancer cells, cancer stem cells and non-cancerous cells [56, 57]. Generally, tumors grow nicely initially and then "stagnate.", and injection of BME/Matrigel near the tumor center will reinitiate growth. Countless lines of tumor cells that do not grow individually in mice can grow, and tumors that are already well-grown grow faster. The higher the concentration of BME/Matrigel, the faster the growth of tumor cells, because the presence of growth factors in Matrigel is conducive to the xenograft of primary tumor cells, and additional growth factors are added to further promote growth [58]. This not only accelerates the growth of tumor cells, saving time and animal costs, but also considerably increases the number of animal models of human cancer. The uptake rate is classically greater than 80%, which is much higher for most cancers [59].

The addition of Matrigel made cell lines more accessible from xenografts, improved the proximity between the tumor spheroid environment and the tumor growth environment in vivo [60]. Many research groups have mixed BME/Matrigel with tumor cells and injected them in vivo to investigate the therapy of tumors, as well as determine whether genetically modified tumor cells could form tumors [61]. BME/Matrigel could also be supplemented with type I collagen in the orthotopic mammary fat pad model to promote the growth and reduce differentiation of breast cancer MCF7 cells [62]. In order to better analyze the effect of Matrigel, Michael with colleagues adopted a bilateral planting method in the PDX-IM model of colorectal cancer: one side was pre-soaked in Matrigel, which significantly ameliorated the tumor extraction rate compared to the remaining side without Matrigel [63]. More definitely, the combination of BME/Matrigel with patient biopsy materials has been commercialized by several companies (Champions Oncology, Oncostat, and Crown Bio) for "precise" or "personalized" drugs.

### Implantation site

The PDX-IM model is divided into ectopic and orthotopic implantation. Ectopic implantation is the implantation of material into an area unrelated to the original tumor site, mostly subcutaneously. Subcutaneous transplantation of PDX-IM models has been widely developed due to its simple operation, high success rate and more accurate monitoring of tumor size [42]. The most common implant site is the dorsal side of the mouse, which is particularly suitable for situations that require large transplants over a short period of time. Alternatively, implantation in the same organ (orthotopic transplantation) as the primary tumor could be selected, such as pancreas, oral cavity, ovary, breast fat pad, brain [44]. Orthotopic transplantation might be an ideal approach, with the advantage that the tumor could develop in the same anatomical microenvironment and thus exhibit more similar behavior to the patient's tumor, especially in terms of metastasis. For several tumor types (such as ovarian, lung and testicular cancers), orthotopic transplantation has significantly increased the incidence of tumors [64]. However, this approach requires trained surgical techniques to generate appropriate PDX-IM models, which are complex and expensive, and imaging techniques are commonly used to monitor tumor growth. Modeling typically takes 2 to 4 months, and failure to transplant is indicated if no tumor growth is observed for 6 months.

Several approaches unrelated to the source of the tumor have implanted primary tumors into the renal capsule to improve the success rate of transplantation. The blood vessels in the subrenal capsule (SRC) site are more abundant compared to the subcutaneous transplantation site, and the fertilization rate of most intact transplanted tissues is high, including benign prostate tissue [47]. The implantation rate of non-small cell lung cancer (NSCLC) in the renal capsule is as high as 90%, while that after subcutaneous implantation is only 25%, and these results are not derived from a single comparative study [65]. Recently, Wu et al. have standardized a transplant protocol and established PDX-IM models of hormone-naive (D17225) and castration-resistant (B45354) PC by implanting fresh tumor samples that obtained from patients with advanced PC under the renal capsule of immune-compromised mice, thus demonstrating the significant effectiveness of the infrarenal zone in the modeling of localized prostate tumors [48]. Furthermore, renal capsule implantation shortens time for engraftment, which is one of the most significant variables in the research of seeking real-time PDX-IMs data for personalized cancer treatment [66].

### Xenograft material

#### Tumor debris or single-cell suspensions

Two different graft materials were utilized for the generation of PDX-IM models, including tumor debris or single-cell suspensions digested by tumors. The application of tumor discrete fragments and single cell suspensions in PDX-IM models has their own characteristics. Tumor debris retains the interconnection between tumor cells and some structural characteristics of the original tumor, thus mimicking the microenvironment of the tumor. Alternatively, the single-cell suspensions could avoid biased sampling of the entire tumor and achieve indiscriminate selection of subclones during analysis or tumor passage. However, single-cell suspensions expose tumor cells to chemical or mechanical forces that may sensitize cells to anoikis, thereby affecting cell survival and transplantation success [67]. Dong et al. established 33 RCC mice xenograft models by tumor debris and single cell suspension with a total implantation rate of 45%, but the success rate of single cell suspension transplantation was 17% different from that of surgical resection of tumor debris transplantation [17]. In addition, the transplanted tissue, typically 1–2mm^3^ in size, needs to be kept fresh during the transplantation process, which means that the time from the operating room to the laboratory should be as short as possible. After the operation, the tissue was immediately preserved in a cold, fresh medium. In a recent study, the ex vivo times of successful cases of gastric cancer PDX-IM differed greatly from unsuccessful cases (median time for successful cases was 75 min vs. 135 min for unsuccessful cases, *P* = 0.003). Similarly, shorter overall procedure time was associated with engraftment success (123 min for successful engraftment vs. 167 min for unsuccessful engraftment, *P* = 0.01) [17]. In addition, the process of sample collection, preservation, and transportation is critical to ensure maximum freshness of samples. This process also takes into account the number of transplanted tissues and the appropriate percentage of tumor cells in the tissues, with the greater the number of fragments, the higher the success rate [67]. There are differences in genome and gene expression levels among different isolates of the same cell line. A cell line represents only one tumor type and actually only one patient in many cases, thus the success rate of transplantation will be relatively changed with different isolates [68]. In one study, Madhavi et al. compared the growth and metastasis of estrogen receptor negative (ER^−^) breast cancer cell lines (MDA-MB-231, SUM1315, CN34BrM) and an ER^+^ cell line (T47D) in immune mice. The results demonstrated that the weight and size of tumors of each cell line were significantly different at different time points, and the mice also showed different signs of pain [69].

#### Surgical excision, biopsy or metastatic samples

In malignant tumors, radical surgical resection is superior to partial resection or biopsy in preserving tumor integrity. This is exemplified in a comparison of transurethral resection of the prostate (TURP) with radical prostatectomy for the treatment of prostate cancer. TURP is more prone to generate tissue debris, leading to the destruction of tissue structure, thereby reducing tumor heterogeneity and reducing tumor cell invasion. In a prostate cancer study, successful PDX-IM models were all developed from tissues derived from radical prostatectomy [70]. Furthermore, Lawrence et al. investigated the factors that determine the initial engraftment of patient tissue extracted from TURP specimens and confirmed that only 21% of the grafts contained cancer at the time of harvest. Grafts were most successful when the original patient specimen contained significant amounts of viable cancers, defined as a specimen with (I) at least 50% cancer cells, (II) no physical damage, and (III) detectable Ki67 expression [71]. In colorectal cancer, katsiampoura et al. found a higher success rate of modeling tumor tissue obtained by surgical resection (36/50 = 72%) than biopsy (14/40 = 35%). In short, specimens resected with surgical integrity would be preferable [72]. However, the existence of a clinical biopsy is indispensable in order to open up xenotransplantation to a broader population of cancer patients in some unresectable primary tumors [73].

PDX-IMs can be successfully created from clinical biopsy specimens that are metastatic or primary, and metastatic cancers exhibit higher engraftment rates. In one study, biopsy specimens from 29 patients were used for engraftment of PDX-IMs, and PDX-IM models created from metastatic biopsies had higher engraftment rates compared with unresectable primary tumor tissue (69 vs. 15.4%, *P* = 0.001) [74]. Masanori et al. established a PDX-IM model of human brain metastases from breast cancer in the mouse brain with an engraftment rate of 100% (10/10) [75]. In a study of colon cancer, the engraftment rate of the PDX-IM models of metastatic tumors was similarly high at 100% (8/8), as compared with 84% (27/32) in primary cancers [76]. A higher engraftment rate was also observed upon engraftment of primary tumors from distant metastases into the PDX-IM models [77]. These data suggest that the ability of tumors to grow continuously in mice is associated with tumor metastasis, due to the fact that metastasized tumors may be more active and invasive. Of course, there is also related to the degree of differentiation of the tumor. The growth rate of metastases is not limited, and PDX-IM models have indeed demonstrated genomic and transcriptomic signatures of metastatic and recurrent carcinomas in some cases [78].

#### Tumor stage and size

Different tumor stages play a crucial role in transplantation rates. It was found that non-small cell tumor samples from stage II (43/96, 45%) and stage III (25/49, 51%) patients showed a higher engraftment rate than stage I (32/145, 22%) [79]. Oh et al. demonstrated that the same results were seen in colorectal cancer xenograft mice, with transplantation rates corresponding to different tumor stages of 4 of 15 (26.7%) stage I tumors, 41 of 72 (56.9%) stage II tumors, and 50 of 84 (59.5%) stage III tumors, and 55 of 70 (78.6%) stage IV tumors [80]. Advanced tumors tended to correspond to larger sized tumor volumes, and hepatocellular carcinoma samples taken from patients with large-sized tumors (> 5 cm) showed a higher engraftment rate of PDX-IMs (87/130, 67%) than those with small-sized tumors (≤ 5 cm) (16/124, 12.9%) [81]. Jung et al. successfully produced 20 PDX-IMs of pancreatic cancer and also found that tumor size was an important factor in the success of PDX-IM [82]. However, tumors are routinely inoculated into mice to grow the next generation of PDX-IM when the primary tumor volume transplanted into F1 generation mice approaches 1000 to 2000 mm^3^ [83, 84]. Excessively large tumors easily affect the survival state of mice, resulting in the lack of nutrition of tumor cells, and the transplantation ability of the tumor is weakened. If the tumor is too small in size, there will be insufficient stromal cells to form the next generation of tumors. The multiple tumor fragments being transplanted are mostly 1–2 mm in diameter [85–87]. Excessive tumor volume easily affects the accuracy of tumor transplantation, leading to a shift in the transplantation position, reducing the transplantation rate. On the contrary, tumor fragments that are too small may not adequately reflect the heterogeneity of the primary tumor, thereby affecting the predictive value of PDX-IM in drug screening.

#### Treatment status

Whether patients treated prior to tumor resection will hinder the successful establishment of PDX-IM models remains controversial. In gastric cancer, 63 PDX-IM models were successfully established from 185 fresh gastroscopic biopsies and maintained in vivo through passage. The results showed that the implantation rate of the biopsy tissues inoculated before chemotherapy (52.1%, 37/71) was higher than that of the biopsy tissues inoculated after chemotherapy (21.9%, 25/114) [88]. Kuwata et al. also found that the success rate of PDX-IM establishment was higher in gastric cancer patients who received chemotherapy than in those who did not (26.4% (9/34) vs. 13.1% (26/198)) [83]. In addition, samples from 133 patients with resected pancreatic duct adenocarcinoma were successfully transplanted into mice, of which 42 samples (32%) received chemotherapy, and the remaining 91 samples (68%) did not [89]. However, in a study of NSCLC PDX-IMs, the engraftment rate without preoperative chemotherapy was 32% (81/247) compared with 37.3% (22/59) in the chemotherapy group, indicating that preoperative chemotherapy did not significantly affect the engraftment rate [15]. The reason may be that part of lung cancer patients treated with chemotherapy have a high degree of tumor differentiation, and even after chemotherapy the malignant tumors are strongly invasive and metastatic. Taken together, chemotherapy may have an impact on the activity of tumor samples, which will reduce the engraftment rate of biopsied tissue. However, this conclusion is not universal across different tumors. The core is to consider the invasiveness, metastasis, as well as the actual situation of the tumor samples.

### Gender gap

With the exception of hormone-dependent prostate and breast cancer, the engraftment rates of PDX-IM models in most other tumor types are independent of the sex of the sample or that of the mouse [90, 91]. However, mouse models of gastric cancer tissue or intestinal gastric cancer tissue from male patients are more likely to be successfully established [92]. The androgen receptor (AR) was demonstrated to directly regulate miR-125b expression and the AR-miR-125b signaling pathway inhibits apoptosis and promotes proliferation, thus may improve transplantation efficiency.

Overall, as these points are handled properly, PDX-IM will be constructed more effectively, which in turn provide more potentially predictive value. There are some critical factors in the establishment of PDX-IM models of partial cancers (Table 5), which still require further study.

**Table 5:** Key points for establishing PDX-IM models in different tumors

Tumor type	Key points
Cholangiocarcinoma	The different genetic backgrounds of recipient mice correlated with transplantation rates
Breast cancer	1. The supplementation of estradiol and Matrigel is necessary; 2. The hormone-dependence is the major limiting factor; 3. The stable take rate of ER- significantly higher than that of ER^+^; 4. Presence of mouse host stroma is required for tumor growth; 5. ER expression was a major determinant of take rate
Pancreatic cancer	1. The differences of pearson correlations may be dependent on tumor type; 2. Tumor size was the significant factor related to successful PDX-IM generation; 3. The rates were higher, when the NOD/SCID or NSG mice were employed
Gastric cancer	1. Prior chemotherapy may reduce the engraftment achievement ratio; 2. Biopsies prior to chemotherapy had a higher transplantation rate than biopsies after chemotherapy; 3. The more severe immunodeficient species may offer a superior platform; 4. GC tissues from male patients or of intestinal subtype were easier to grow up in mice; 5. Ex vivo time and overall procedure time were the significant
Colorectal cancer	1. The epithelial subtypes, the largest subgroups of CRC subtype, were very ineffective in establishing PDX-IMs; 2. The major subtype CMS2 is strongly underrepresented in PDX-IM; 3. Micro tumor tissues with sizes ˂ 150 μm in diameter were more fitted to maintain the tumor microenvironment
Lung cancer	1. The engraftment can be affected by the histological subtype, the immune microenvironment, and the lymphoma formation; 2. Positive engraftment correlating with shorter disease-free survival in a multivariate analysis including age, sex, stage, and mutations; 3. The main deterrent in engraftment success is likely tumor cellularity in these small TBNA samples
Ovarian cancer	1. The quality of patient tumor tissues, location of implantation site, and type of immuno-deficient mice are possible factors responsible for successful engraftment; 2. Concomitant administration of estradiol pellets in the contralateral flank for SC transplants; 3. Compared to EOC, the take rate of nonepithelial ovarian cancer seemed to be higher
Head and neck cancer	1. Biopsy showed a significantly higher engraftment rate compared to surgical resection; 2. Metastatic sites showed a significantly higher engraftment rate compared to primary sites; 3. HPV positivity tends to show a low engraftment rate; 4. Outgrowth of EBV + lymphomas is a potential barrier to durable engraftment of HPV + HNSCCs
Glioblastoma	The success rate was lower than other tumors
Prostate cancer	1. Prostate cancer xenografts are prone to be outgrown by early EBV-positive lymphomas; 2. To establish a PC PDX-IM, the most critical step is access to tissues of good quality and viability
Melanoma	The success rate of PDX-IM has significant bias toward BRAF, TP53 mutations and CDKN2A loss
Renal cell carcinoma	1. Higher stage, grade, and sarcomatoid differentiation were among the parameters that favor engraftment; 2. The correlation between stable engraftment in mice and poor survival; 3. The viability and stability of using biopsy tissue to generate xenograft models
Cervical Cancer	Ervical dysplasia and normal cervical tissue can generate microscopic tissues in the PDX-IM model
Malignant Pleural Mesothelioma	PDX-IM models of MPM can be derived from all histologically subtypes and from small biopsy specimens

## PDX-IM models in cancer research

### Screening of drugs and diagnosis of biomarkers

**Fig. 3:**
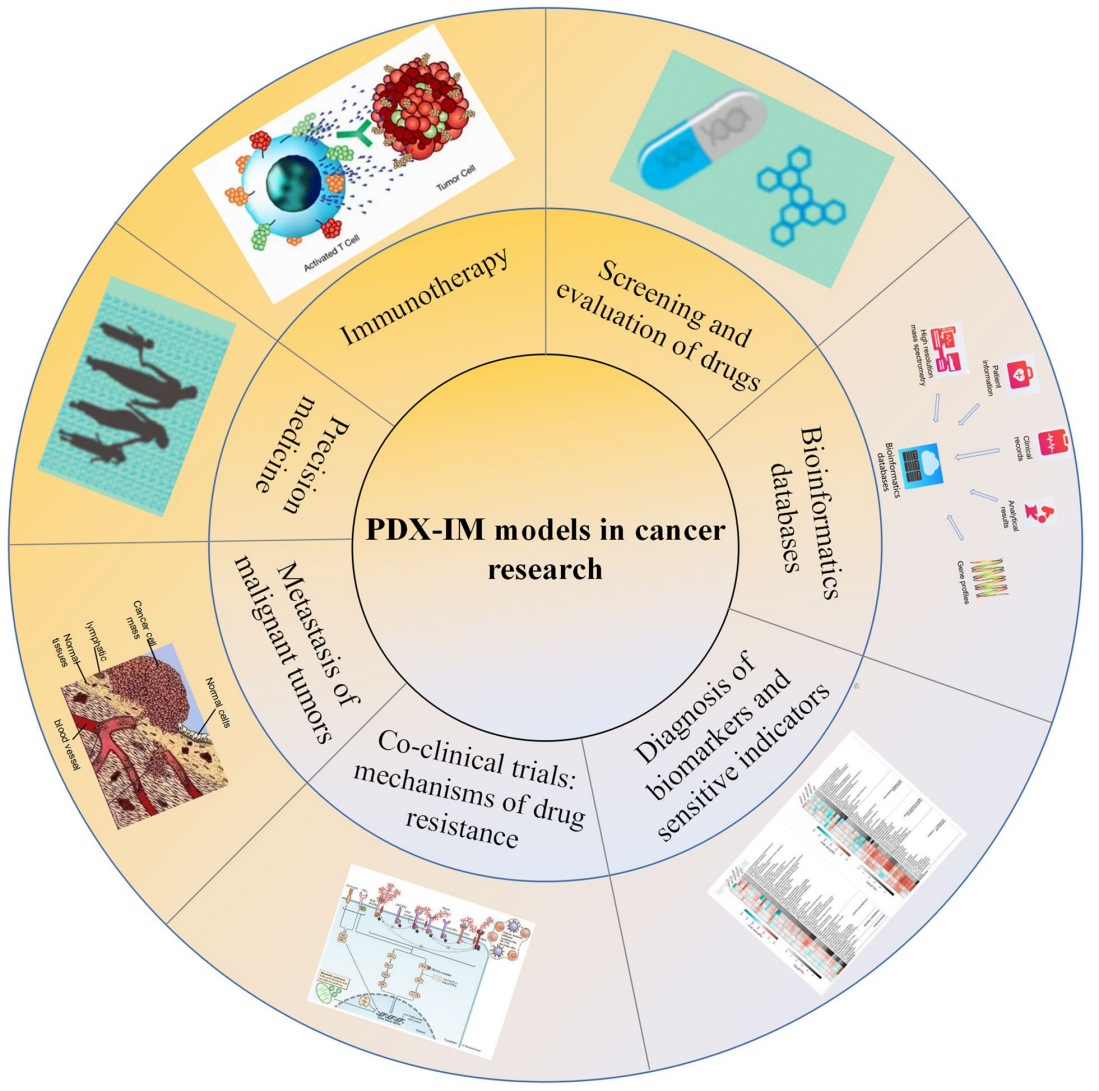
PDX-IM applications in cancer therapy, such as screening and evaluation of drugs, bioinformatics databases, diagnosis of biomarkers and sensitive indicators, co-clinical trials (mechanisms of drug resistance), metastasis of malignant tumors, precision medicine, and immunotherapy

PDX-IMs are substantial for clinical decision-making before human clinical trials, the development of anti-cancer agents, and diagnosis of biomarkers (Fig. 3). One of the major problems in oncology drug development is the low success rate of new drugs, with only 5% of preclinical anticancer drugs eventually approved for clinical treatment. Many anticancer drugs failed due to lack of efficacy in phase II and III clinical trials and wasted a lot of resources, mainly because of the low predictive value of conventional preclinical models for screening new formulations for clinical development [39]. As a preclinical model with high predictive value, the PDX-IM model plays an irreplaceable role in preclinical screening of new anticancer drugs. In the absence of appropriate biomarkers to detect patient selection and response monitoring for new drugs, PDX-IM models could alter this status quo, both for targeted drugs and for classical cytotoxic drugs. Studies have demonstrated that the drug response rate of PDX-IM model in breast cancer, renal cell carcinoma, non-small cell lung cancer, head and neck squamous cell carcinoma, colorectal cancer and other cancers is very similar to the clinically observed effective rate [67].

In addition to identifying potential clinical indications, PDX-IM models have contributed greatly to the diagnosis of potential biomarkers. In fact, PDX-IMs provide a virtually unlimited source of tumor tissue for multi-dimensional molecular profiles and allow detecting responses to multiple drugs in the same model. According to the consistency between PDX-IM model and human trials, the corresponding drug biomarkers were screened out. In one study, PDX-IM models of colorectal cancers treated with an epidermal growth factor receptor inhibitor, cetuximab, showed comparable response rates to those of the patients in whom the tumor originated [127]. Because of the consistency of drug response in PDX-IM model with clinical practice, the analysis aims to identify tumor-specific and matrix-specific biomarkers, and ultimately to achieve the discovery of drug efficacy research and specific biomarkers [128].

### Co-clinical trials and precision medicine

Once a drug enters clinical trials, the opportunity to analyze and integrate useful information for the development of the formulation on a real-time basis is limited [129]. In the absence of comprehensive and more in-depth clinical observations, patients may experience extreme responses or rapid drug resistance, and thus the concept of co-clinical trials has been proposed to address these issues. Co-clinical trials refer to concurrent preclinical and clinical trials (parallel studies between mouse models and patients) that comprehensively analyze and integrate relevant clinical, biological and pharmacological information to identify predictive biomarkers for specific therapeutic responses, even in rare types of cancer. These studies initially applied the results of genetically engineered mouse models to clinical trials and were validated in the treatment of acute promyelocytic leukemia. This trial, considered a model of personalized care or precision medicine [130], has shown positive results in parallel clinical trials, including clear cell adenocarcinoma [131], melanoma [132], oral squamous cell carcinoma [133] and small cell lung cancer [134]. For example, the PDX-IM model of 85 patients with metastatic colorectal cancer was established to predict resistance to targeted anti-EGFR therapies using a combination of anti-HER-2 and anti-EGFR therapies. The results suggest that patients with metastatic colorectal cancer resistant to cetuximab and those with concomitant HER-2 amplification and ineffective clinical treatment may benefit from the combination of HER-2 inhibitors and EGFR inhibitors [135].

Most human solid tumors did not metastasize after subcutaneous implantation in nude mice, whereas the PDOX (Patient-derived orthotopic xenograft) model could recapitulate the local aggressive growth as well as metastasis behavior of primary tumors, and is commonly used to establish a metastatic tumor model. Metastasis models typically involve orthotopic transplantation of primary tumor fragments waiting for transplanted tumor growth to produce spontaneous metastasis [136], or direct orthotopic transplantation of metastases [137]. Drug sensitivity may differ between primary and metastatic tumors grown in the PDOX model, which were previously undetectable in the subcutaneous graft tumor model. For example, neither the subcutaneous PDX-IM model nor the PDOX model of HER-2-positive cervical cancer nude mice were sensitive to the benzamide histone delactase inhibitor eninostat, but in the PDOX model, the drug significantly reduced the load of metastatic tumors compared with the control group [138]. PDOX model could better reflect the biological process of tumor metastasis by retaining the microenvironment of tumor, including the role of stroma in the process of tumor treatment, the mechanism of tumor metastasis, the drug response of metastatic tumors and other related clinical studies [139], which is of great significance to the precise treatment of tumor metastasis.

Oncology research has evolved in parallel with the improved understanding of the cancer genotype and phenotype. Multiple potential targets have been identified in some patients, making it difficult to select the most appropriate target, ushering in a new era of precision medicine. Different from traditional chemotherapy, precision medicine combines the characteristics of individual patients, that is, investigating the genomic profiles of tumors through molecular targeted drugs or immunotherapy to maximize therapeutic efficacy and minimize side effects [140]. Practically, the concept of precision medicine is to divide patients into different Gene subpopulations based on sophisticated genomic profiling, enabling certain therapies to target specific subgroups [42]. In view of this, PDX-IM models, which play an increasingly important role in personalized medicine, not only represent subpopulations with similar genetic profiles, but also recapitulate the intratumoral heterogeneity of tumors in primary patients (Table 6). Meanwhile, its genomics, metabolomics and microbiome analysis are the closest to entering clinical practice [141].

**Table 6:** Application of PDX-IM model in preclinical or clinical studies of several common tumors

Tumor type	Drug name	Implantation site	Therapeutic target	Application	References
Lung cancer	Gefitinib	Subcutaneous	EGFR	Drug resistance mechanism study	[142]
	ASK120067	Subcutaneous	EGFR	Novel drug validation	[143]
	GSK2849330	Subcutaneous	HER3	Drug combination validation	[144]
	HER3-DXd	Subcutaneous	HER3	Novel drug preclinical validation	[145]
	Gefitinib	Subcutaneous	EGFR	Drug resistance mechanism study	[146]
Breast cancer	AZD4547, BLU9931	Mammary fat pad	FGFR1, FGFR2, FGFR4	Therapeutic target identification	[147]
	BYL-719, selumetinib	–	PI3K, MEK	Drug combination validation	[148]
	BAY80-6946, PF-04691502, AZD2014	–	PI3K p110α subunit, mTOR and PI3K, mTORC1 and mTORC2	Therapeutic target identification	[149]
	MLN0128, trastuzumab	Mammary fat pad	dual mTOR complex, HER2	Drug combination validation	[150]
	U3-1402	Subcutaneous/mammary fat pad	HER3	Novel drug validation	[151]
	Pan-HER	Mammary fat pad	Pan-HER antibody mixture against EGFR, HER2, and HER3	Drug combination validation	[152]
	Docetaxel, 5-fluorouracil, Trastuzumab	Mammary fat pad	–	Novel drug validation	[153]
	Docetaxel, doxorubicin, trastuzumab + Lap	Mammary fat pad	–	Novel drug validation	[68]
Pancreatic ductal adenocarcinoma	Trametinib	Subcutaneous	MEK	Drug combination validation	[154]
	Gemcitabine	Heterotopic	–	Drug resistance mechanism study	[155]
Ovarian cancer	Pertuzumab/trastuzumab	Intraperitoneal (IP) injection	HER2	Drug combination validation	[156]
	Cisplatin	Heterotopic	–	Drug resistance mechanism study	[157]
Pancreatic tumor	Compound 36 l	–	KRAS‒PDEδ	Novel drug preclinical validation	[158]
	Palbociclib, Trametinib	Subcutaneous	CDK4/6, MEK	Drug combination validation	[159]
Gastric cancer	Avapritinib	–	Mutated KIT	Novel drug preclinical validation	[160]
	Lenvatinib	Subcutaneous	Multitargeted tyrosine kinase inhibitor	Novel drug preclinical validation	[161]
	Regorafenib	Subcutaneous	VEGFR, MVD	Novel drug preclinical validation	[162]
Colorectal cancer	Cetuximab, LSN3074753	–	EGFR, RAF	Drug combination validation	[163]
	Cetuximab, Panitumumab	Subcutaneous	HER2	Therapeutic target identification	[135]
	WT KRAS	Subcutaneous	IGF2	Therapeutic target identification	[164]
	Oxaliplatin	Heterotopic	–	Drug resistance mechanism study	[165]

### Immunotherapy and bioinformatics databases

In recent years, immunotherapy has achieved widespread success against a variety of malignancies. The humanized PDX-IM model facilitates the study of tumor biology and immune system function by reconstructing the human immune system and tumor growth. Zhao et al. developed a PDX-IM model matching the human immune system as an immuno-oncology model using NOD-SCID Il2rg (NSG) mice and investigated immunotherapy approaches utilizing type I humanized leukocyte antigen in mice. Among them, the treatment and side effects of phenylpropanolizumab and ipilimumab have been investigated in this model [35]. In cell therapy, PDX-IM models can be used to evaluate various aspects of CAR-T cell therapy and biology. In particular, for the interaction between CAR-T and other immune cells (such as Tregs and bone marrow derived suppressor cells (MDSC)) in the tumor microenvironment, PDX-IM models will show more accurate and acceptable results [166]. The humanized PDX-IM model is a future tool for personalized medicine that will support clinical decision-making. In an avatar of human melanoma patients (hIL2-NOG mice), anti-PD-1 (programmed cell death protein 1) antibody responses and tumor-infiltrating T cells support clinical decision making for immunotherapy [167]. However, the humanized immune PDX-IM model still needs more validation.

Many institutions and organizations were committed to creating a large number of PDX-IM or PDX-IM bioinformatics databases [139]. These bioinformatics databases with patient clinical data, pathology, gene profiles and drug response data are essential for predicting and validating drug response information from tumors with similar genetic backgrounds. The successful establishment of a global PDX-IM bioinformatics database has contributed to the rapid acquisition of similar PDX-IM models by comparing data related to specific patients, thereby transforming traditional clinical treatment concepts and facilitating the transition from individualized to programmed therapies. By comparing clinical samples with those in the database, the optimal therapy regimen can be determined from a shared database when patient genomic characteristics are similar or consistent [44, 168]. Currently, PDX-IM bioinformatics databases are available in the United States and Europe (Table 7), and most PDX-IMS are derived from common cancers [139, 169]. Similarly, PDX-IM bioinformatics databases in Asia and PDX-IM for rare cancers are indispensable, which facilitates the sharing between global PDX-IM bioinformatics databases, as well as the popularization of PDX-IM across all tumors.

**Table 7:** PDX-IM bioinformatics databases

Region	Bioinformatics databases	Cancer type
Europe	Luxembourg Institute of Health	Glioma
	Vall d’Hebron Institute of Oncology	Breast carcinoma, pancreas cancer, colorectal cancer
	Candiolo Cancer Institute	Gastric cancer and colorectal cancer
The United States	St. Jude Children’s Research Hospital	Pediatric solid tumors
	Pediatric Preclinical In Vivo Testing Consortium	Pediatric Pan-cancer
	Washington University in St. Louis	Pan-cancer
	Charles River Laboratories	Pan-cancer
	The Center for Patient Derived Models at Dana Farber Cancer Institute	Pan-cancer
	NCI Patient-Derived Models Repository	Pan-cancer
Canada	Princess Marget Living Biobank	Pan-cancer

## Challenges and prospects in PDX-IM models

The ability to directly transfer human tumors into mice and perform multiple in-vivo passages provides unique opportunities for cancer research and drug discovery, making PDX-IM a valuable cancer model. However, like other model systems, understanding the limitations is necessary for optimal application [170]. Firstly, the longer time of model establishment limits the application in patients with a shorter expected survival [67]. Second, the establishment and maintenance are costly, and the amount of tissue available for implantation is limited. In order to improve transplantation rates, the next phase of PDX-IMs development aims to identify the most appropriate conditions and methodologies to maximize tumor formation [3], sometimes requiring transplantation of smaller samples for personalized medication, such as fine needle aspiration. Third, the incidence of developing EBV-related B-cell lymphoma was as high as 68% when PDX-IM models were generated using severe combined immunodeficient mice NOD/SCID, NSG, or NOD, especially in the F1 generation (33.3%) [17]. However, lymphomagenesis can be reduced using nude mice, which do not form lymphoma even when NOD (F2) mice are used in subsequent transplants. Fourth, the pharmacodynamic evaluation system of the PDX-IM model needs to be improved. Tumor growth retardation during therapy typically results in a larger tumor volume at the end point than before treatment, but smaller than in the control group, indicating that the treatment is biologically active. In fact, this response does not imply clinical efficacy, but is only clinically defined as "disease progression" or even "disease stabilization" [139], requiring quantitative indicators to categorize responses in order to more accurately assess treatment effects in trials. Some researchers have proposed improved evaluation criteria [168], which combines reaction speed, intensity and persistence to further unify the interpretation of treatment response. Fifth, human stromal components are rapidly lost during implantation and replaced by the microenvironment of mice [171], thus genetic heterogeneity cannot be fully manifested in dissected tumors of passage, which results in tumors with genetic heterogeneity that cannot always be reproduced in successive passages. PDX-IM models have been reported to undergo mouse-pecific tumor evolution with rapid accumulation of copy number alterations during PDX-IM passaging, which differed from those acquired during tumor evolution in patients by the strong selection pressures in the mice [170]. Consequently, PDX-IMs need to find a solution in the context of simulating a fully accurate human tumor microenvironment. In this respect, advanced real-time imaging systems are able to quantitatively assess the growth and metastatic progression of primary tumors. Bioluminescence imaging of PDX-IMs from organs is a highly sensitive approach for detecting micrometastasis lesions, but relies on the use of imaging modalities [172, 173]. Finally, further research is necessary to develop strategies for evaluating the efficacy of immunosuppressive checkpoint inhibitors, as the PDX-IM model was established only in immunodeficient mouse strains. Long-term preservation of PDX-IM models is difficult and requires the establishment of efficient long-term cryopreservation conditions to prevent microbial infection. Previous studies have shown that special cryoprotectants exhibit superior performance over traditional media [174].

## Conclusion

Different tumor molecular signatures correspond to different therapeutic responses, which are not well represented in most preclinical models. Since the advent of the first tumor models, PDX-IM models have demonstrated significant tumor heterogeneity and are among the most reliable and standard models in preclinical studies. In addition, as a promising and innovative preclinical tool, PDX-IMs are available for the study of tumor initiation, progression, and metastasis (generally orthotopic transplantation). However, despite the increasing relevance of PDX-IM models in cancer research and treatment, patient-derived models also suffer from limitations due to the lack of human immune cells and stromal cells, which contribute to tumor progression by interacting with tumor cells. We set up a humanized PDX-IM mouse model to recapitulate immune cell interactions in the human tumor microenvironment. It is important to note that, while emphasizing the individualization of tumor PDX-IM models, targeted therapies based on the genomic characteristics of PDX-IM models are required to improve the efficiency of the model's application and even to obtain more tumor subtypes and more effective targeting options. Meanwhile, the confounding factors that affect the efficiency of model establishment need to be considered as well as addressing these key issues: (1) improving the engraftment success rate of the models, (2) accelerating the generation rate, (3) ameliorating long-term preservation conditions, (4) reducing microbial infection, (5) perfecting the pharmacodynamic evaluation system, (6) promoting the application of matrix and immune system related research, etc., is conducive to making PDX-IMs the mainstream model for studying tumor biology, investigating genetic heterogeneity and therapeutic targets. Although there are certain limitations of such models, they hold promise for developing more applications in cancer research.

## Data Availability

All data generated or analyzed during this study are included in this manuscript and its Additional file.
